# Identification of miRNA-Mediated Subpathways as Prostate Cancer Biomarkers Based on Topological Inference in a Machine Learning Process Using Integrated Gene and miRNA Expression Data

**DOI:** 10.3389/fgene.2021.656526

**Published:** 2021-03-24

**Authors:** Ziyu Ning, Shuang Yu, Yanqiao Zhao, Xiaoming Sun, Haibin Wu, Xiaoyang Yu

**Affiliations:** ^1^The Higher Educational Key Laboratory for Measuring and Control Technology and Instrumentations of Heilongjiang Province, Harbin University of Science and Technology, Harbin, China; ^2^School of Medical Informatics, Daqing Campus, Harbin Medical University, Daqing, China

**Keywords:** machine learning, SVM, cancer, topological information, miRNA-mediated subpathway

## Abstract

Accurately identifying classification biomarkers for distinguishing between normal and cancer samples is challenging. Additionally, the reproducibility of single-molecule biomarkers is limited by the existence of heterogeneous patient subgroups and differences in the sequencing techniques used to collect patient data. In this study, we developed a method to identify robust biomarkers (i.e., miRNA-mediated subpathways) associated with prostate cancer based on normal prostate samples and cancer samples from a dataset from The Cancer Genome Atlas (TCGA; *n* = 546) and datasets from the Gene Expression Omnibus (GEO) database (*n* = 139 and *n* = 90, with the latter being a cell line dataset). We also obtained 10 other cancer datasets to evaluate the performance of the method. We propose a multi-omics data integration strategy for identifying classification biomarkers using a machine learning method that involves reassigning topological weights to the genes using a directed random walk (DRW)-based method. A global directed pathway network (GDPN) was constructed based on the significantly differentially expressed target genes of the significantly differentially expressed miRNAs, which allowed us to identify the robust biomarkers in the form of miRNA-mediated subpathways (miRNAs). The activity value of each miRNA-mediated subpathway was calculated by integrating multiple types of data, which included the expression of the miRNA and the miRNAs’ target genes and GDPN topological information. Finally, we identified the high-frequency miRNA-mediated subpathways involved in prostate cancer using a support vector machine (SVM) model. The results demonstrated that we obtained robust biomarkers of prostate cancer, which could classify prostate cancer and normal samples. Our method outperformed seven other methods, and many of the identified biomarkers were associated with known clinical treatments.

## Introduction

Prostate cancer is the second most commonly diagnosed cancer among males worldwide, and it is associated with miRNA dysfunction (Dankert et al., 2020). Prostate cancer is a highly heterogeneous disease with various mutations and tumor cell phenotypes (Peitzsch et al., 2020). The heterogeneity of prostate cancer causes difficulty regarding diagnosis and prognosis. Regarding treating prostate cancer patients, it is hoped that personalized medicine can be developed to mitigate the issues caused by the huge variations between different patient subgroups (Peng et al., 2017; Zhou et al., 2019).

The aim of this study was to identify robust biomarkers associated with prostate cancer, in the form of miRNA-mediated subpathways (miRNAs), and to evaluate the performance of our machine learning method based on other cancer datasets in addition to prostate cancer datasets. To identify cancer-related miRNAs to aid diagnosis and prognosis, high-throughput miRNA expression profiling has been used (Jay et al., 2007; Martens-Uzunova et al., 2012). Many studies have shown that miRNAs are stable not only in bodies but also in paraffin blocks (Baker, 2010). As miRNAs are promising biomarkers for cancer classification, several methods have been proposed to identify cancer biomarkers based on miRNA expression profiles, such as instance-based methods (Breiman et al., 1984; Breiman, 2001) and feature-based methods (Zararsiz et al., 2017; Peng et al., 2018). However, the performance of miRNA classification biomarkers in test sets varies greatly, even among patients with the same disease phenotype. Several factors, such as tissue heterogeneity, racial differences, and sequencing errors, contribute to this problem (Ning et al., 2019).

Many cancer-related pathways can be utilized as important classification biomarkers (Chen et al., 2021). For diagnosis prediction, pathway topological analysis can be used to identify risk classification biomarkers. Therefore, we integrated multiple types of data to identify the key miRNA-mediated subpathways of prostate cancer, which included data on the expression levels of miRNAs and their target genes and the topological weight of each gene in a global directed pathway network (GDPN). We employed a support vector machine (SVM)-based method to identify accurate risk biomarkers of prostate cancer based on the topological inference of miRNA-mediated subpathway activity. The method included five steps: merge pathways and construct network; perform directed random walk (DRW) (Liu et al., 2013); infer miRNA-mediated subpathway activity; select features and evaluate classification method; and obtain risk biomarkers. First, we obtained a dataset from The Cancer Genome Atlas (TCGA), a Gene Expression Omnibus (GEO) dataset, and a GEO cell line dataset, which together comprised 775 normal and human prostate cancer samples. Moreover, we also identified miRNA–target gene pairs in the TarBase v8.0 (Karagkouni et al., 2018) miRTarBase (Chou et al., 2018), and miRecords (Xiao et al., 2009) databases. Additionally, data on 4,090 samples in 10 other cancer TCGA datasets were downloaded from UCSC Xena. Thereafter, 343 Kyoto Encyclopedia of Genes and Genomes (KEGG) pathways were merged into the GDPN, in which the nodes represented genes. Next, the method involved inferring the miRNA-mediated subpathway activity profile using a DRW-based method. Risk classification biomarkers (i.e., the high-frequency miRNA-mediated subpathways) were then identified using an SVM approach. Subsequently, we performed *within-dataset* analyses using the three prostate cancer datasets, and we identified the high-frequency miRNA-mediated subpathways in order to divide the samples into normal and cancer groups. We then evaluated the classification performance of these risk biomarkers in *cross-dataset* analyses using the prostate cancer datasets, followed by evaluating the performance in 10 other cancer datasets.

## Materials and Methods

**FIGURE 1:**
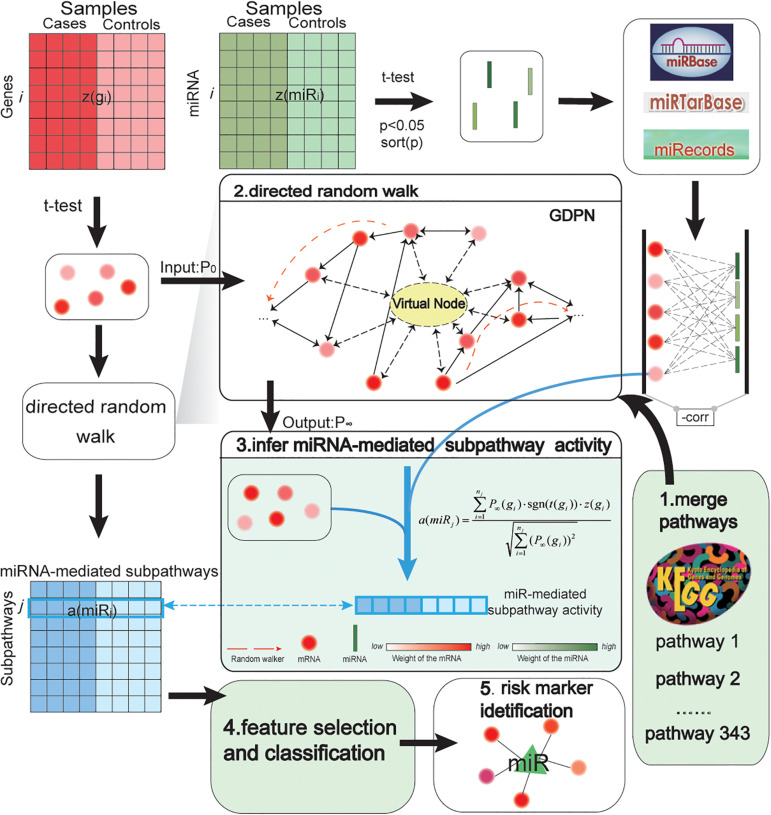
Pipeline showing how miRNA-mediated subpathway activity profiles were inferred based on gene and miRNA expression and topological weights in a global directed pathway network (GDPN). The method to identify accurate risk biomarkers of prostate cancer involved five steps: merge pathways and construct network; perform directed random walk; infer miRNA-mediated subpathway activity; select features and evaluate classification method; and obtain risk biomarkers. *z*(*g*_*i*_) refers to a row-normalized vector of the expression of the *i*th gene over all samples, while *a*(*miR*_*j*_) refers to a row-normalized vector of the activity values of the *j*th miRNA (namely, the *j*th miRNA-mediated subpathway) over all samples. The middle part of the figure shows how the activity profiles were inferred. The 343 canonical KEGG pathways were merged into the GDPN. There are 39,930 directed edges and 7,159 nodes besides the virtual node in the GDPN. The virtual node is represented as a circle with a dotted line. *P*_0_ refers to the matrix of the initial weights of all genes in the GDPN; *P*_∞_ refers to the final weights of all genes in the GDPN. To identify the important upstream genes, regarding assessing the direction of edges between genes, a gene was considered important if it influenced more downstream genes. The expression level and topological weights of the genes are integrated into *a*(*miR*_*j*_).

An overview of our biomarker identification method is shown in Figure 1. The method involves five major steps: merge pathways and construct network; perform DRW; infer miRNA-mediated subpathway activity; select features and evaluate classification method; and obtain risk markers. In addition, we transformed the gene expression profiles into an expression matrix, and we did the same for the miRNA expression profiles. In an expression matrix, each row refers to a miRNA/gene and each column refers to a sample. Next, we integrated the gene and miRNA expression data, the topological weights in the GDPN, and the miRNA–target gene pairs into an activity value. Consequently, we inferred an activity profile, in which each row and each column referred to one miRNA-mediated subpathway (miRNA) and one sample, respectively. After identifying biomarkers, the performance of our SVM model was evaluated using two validation GEO prostate cancer datasets and 10 other cancer datasets.

### Sample-Matched Datasets

We obtained three prostate cancer datasets, each of which included sample-matched gene and miRNA expression profiles. We downloaded one dataset (“PRAD-TCGA”) from UCSC Xena^1^, which involved sample-matched Illumina HiSeq level 3 gene and miRNA expression profiles. After removing the rows in which the expression values were equal to 0, we transformed all gene symbols to Entrez gene IDs. This resulted in 546 samples (52 normal and 494 cancer samples) with 12,118 genes and 209 miRNAs. To conduct an unbiased assessment of the performance of our method, we downloaded an independent dataset (GSE21036) (Taylor et al., 2010), which contained gene and miRNA expression profiles that were obtained using the GPL8227 microarray platform, from the GEO database^2^. We processed this dataset in the same way as the “PRAD-TCGA” dataset. It contained sample-matched data of 139 samples (28 normal and 111 cancer samples) with 18,941 genes and 373 miRNAs. To supplement this small independent validation GEO dataset, we downloaded a sample-matched cell line GEO dataset (GSE14794) on prostate cancer, which contained data that were detected using the GPL6102 and GPL8178 platforms. We used 90 samples (45 control and 45 cancer samples) with 13,935 genes and 273 miRNAs (and no duplicates) from this dataset. Lastly, 10 other cancer TCGA datasets were downloaded from UCSC Xena, which involved 4,090 samples.

### miRNA–Target Genes Associated With Prostate Cancer

To identify the precise local subpathway regions associated with prostate cancer, we obtained reliable miRNA–target gene pairs from the following databases: TarBase v8.0 (Karagkouni et al., 2018), miRTarBase (Chou et al., 2018), and miRecords (Xiao et al., 2009). After removing the duplicates, there were 346,349 human-specific pairs, which consisted of 59 pairs from miRecords, 135,125 from TarBase, and 319,637 from miRTarBase. The specific interactions included two types of target relationships, which had been predicted based on calculations and verified by experiment.

### GDPN Construction

We obtained 343 canonical pathways from the KEGG database based on annotations of the differentially expressed target genes of the differentially expressed miRNAs. We then used the pathway interactions in the KEGG database to create a directed graph, and we merged this into a GDPN using “SubpathwayMiner” software^3^ (Li et al., 2009). If genes appeared in diverse pathways, we merged them and kept the topological graphs. Finally, the GDPN included 39,930 directed edges and 7,159 gene nodes. Each edge direction could be traced back to the type of interaction between the pair of gene nodes according to the KEGG database, i.e., if gene P inhibited/activated gene Q, the edge direction pointed to gene Q. To ensure node weights flow in the network, we added a virtual node to the GDPN, with each node pointing to the virtual node and the virtual node pointing to all the nodes in the GDPN. We confirmed that the distributions of the GDPN node degree approximately followed power-law distributions, with *R*^2^ = 0.72 (in-degree), 0.77 (out-degree), and 0.71 (total degree). Our method obeyed an important rule of the DRW algorithm, which involved having a low proportion of nodes that had higher degrees in the network (Watts and Strogatz, 1998).

### Performing DRW on the GDPN

The DRW algorithm simulated a walker that started at a source node and randomly stayed at the source or traveled to its neighbor node (Liu et al., 2013). New topological weights for the nodes in the GDPN were reassigned using the DRW algorithm, which is similar to the PageRank algorithm (Brin and Page, 1998). The PageRank algorithm is used by the Google search engine to search for related webpages; the higher the number of linkages that are directed toward a webpage, the more important it is. However, in our DRW algorithm, the direction of the linkages was reversed when compared to their direction in the PageRank algorithm, i.e., a gene that influenced more downstream genes was considered more important (Draghici et al., 2007). To calculate the new weights, the standard formula of the DRW algorithm was as follows:

(1) $$P_{t+1}=\left(1-r\right)M^{T}P_{t}+rP_{0}$$

where *M*^*T*^ is a row-normalized adjacency matrix (each element is divided by the sum of all elements in a row); *r* ∈ [0,1] is the restart probability (*r* was set to 0.7), which slightly affected the result of the DRW algorithm (Kohler et al., 2008; Lv et al., 2015); and *P*_0_ is a unit vector of the initial probabilities, which equaled |*t*−*score*| (absolute value), generated based on *t*-test of normal vs. cancer samples. To start the process, *P*_0_ was assigned to each GDPN node (the initial probability of the virtual node equaled 0) and several iterations were required until |*P*_*t* + 1_−*P*_*t*_| ≤ 10^−10^. Eventually, *P*_*t*_ converged to a stable state *P*_∞_, which was a vector of the new topological weights and was considered to represent the GDPN topological information.

### Inferring the Activity Profile From Gene Expression and GDPN Topological Information

For each differentially expressed miRNA between the normal and cancer samples, we determined their target genes and analyzed the differences in expression between the normal and cancer samples. Only target genes that were significantly differentially expressed (*t*-test *p*-value < 0.05) were used to infer the activity value of the miRNA-mediated subpathways. The significantly differentially expressed target genes {*g*_1_, *g*_2_, ⋯, *g*_*n*_*j*__} of miRNA *j* (*miR*_*j*_) were incorporated into an activity value, namely, miRNA-mediated subpathway activity *a*(*miR*_*j*_). In light of this, we have the following:

Constraint:

(2) $$t\left({\mathrm{miR}}_{j}\right)\cdot t\left(g_{i}\right)<0$$

(3) $$a\left(miR_{j}\right)=\frac{\sum_{i=1}^{n_{j}}P_{\infty }\left(g_{i}\right)\cdot sgn\left(t\left(g_{i}\right)\right)\cdot z\left(g_{i}\right)}{\sqrt{\sum_{i=1}^{n_{j}}{\left(P_{\infty }\left(g_{i}\right)\right)}^{2}}}$$

where *t*() is |*t*−*score*| of miRNAs or genes based on a *t*-test between the normal and cancer samples; *sgn*() is a sign function {if *sgn*[*t*(*g*_*i*_)] is equal to a positive number, *sgn*() returns +1, otherwise, it returns –1}; *z*(*g*_*i*_) is the normalized expression vector of gene *g*_*i*_; *P*_∞_(*g*_*i*_) is the topological weight obtained by DRW; *a*(*miR*_*j*_) is the *j*th miRNA of the activity; and *n*_*j*_ is the total number of significantly differentially expressed target genes. For Equation 3, Equation 2 is a constraint that ensures an inverse correlation between the expressions of miRNAs and their target genes. For example, to calculate the activity value of downregulated miRNAs, we integrated the expression of their upregulated target genes and the topological weights of their upregulated target genes into a special value. For upregulated miRNAs, we used the same method to calculate miRNA-mediated subpathway activity. Thus, the rows and columns indicated the miRNA-mediated subpathways (miRNAs) and samples, respectively and each value in the activity profile referred to the activity level of one miRNA in one sample.

### Evaluating Classification Performance

We performed fivefold cross-validation in *within-dataset* analyses of the “PRAD-TCGA,” GSE21036, and GSE14794 datasets. In each of the three *within-dataset* analyses, we randomly split the samples into five equal parts and selected four for training (*training* set) and one for testing (*test* set). Furthermore, the *training* set was randomly split into three equal parts, of which two (*training* subset) were used to build the classifiers and select candidate features, and the remaining one (*test* subset) was used to optimize the classifiers and select the risk biomarkers. First, we used a Student’s *t*-test to obtain the *p*-values of the differences in the miRNA-mediated subpathway activities between the normal and prostate cancer samples in the *training* subset, and we sorted them by ascending *p*-value. We used the top 50 miRNA-mediated subpathways of the *training* subset as candidate biomarkers to establish the first classifier. The first classifier was built based on the candidate biomarker with the smallest *p*-value. Then, we added the candidate biomarker with the second-ranked *p*-value to it. If the area under the curve (AUC) increased, this candidate biomarker was kept in the risk biomarker set; otherwise, it was removed. We performed this process 50 times. We obtained the first optimized classifier and the average AUC from the first *test* subset. Thus, we could obtain three optimized classifiers from three *test* subsets. Then, we evaluated each of the three optimized classifiers by the five *test* sets in turn. We could obtain 15 AUCs. The experiment was repeated 10 times in each *within-dataset* analysis. We obtained the average AUC among the resulting 150 classifiers, which was used to represent the overall performance of our SVM-based method. Each SVM model was built using the “e1071” package in R (Meyer, 2013), which provides an R interface to libsvm. The functions “svm()” and “predict()” were used to build each SVM model and to predict the sample types, respectively. The “e1071” package was also used to perform the evaluation in the *cross-datasets* analyses.

Regarding the two *cross-datasets* analyses (“TCGA–GSE21036” and “TCGA–GSE14794”), we performed fivefold cross-validation, with the “PRAD-TCGA” dataset being used as the *training* set and GSE21036 or GSE14794 being used as the *test* set. We split the “PRAD-TCGA” samples into five equal parts and selected four for training (*training* subset) and the remaining one for testing (*test* subset). The validation process was similar to that in the *within-dataset* analyses. The *training* subsets were used to build the classifier and provide candidate biomarkers, and the *test* subset was used to optimize the classifier and select risk biomarkers. In each of the two *cross-datasets* analyses, five classifiers were optimized (optimizing the AUCs) by five *test* subsets in turn. The final performances of these classifiers were tested on the *test* set. For an unbiased assessment of the performance of our method, each validation experiment (“TCGA–GSE21036” and “TCGA–GSE14794”) was repeated 10 times, and the average AUC was generated among the resulting 50 classifiers.

## Results

### Inferred miRNA-Mediated Subpathway Activity Profile

**FIGURE 2:**
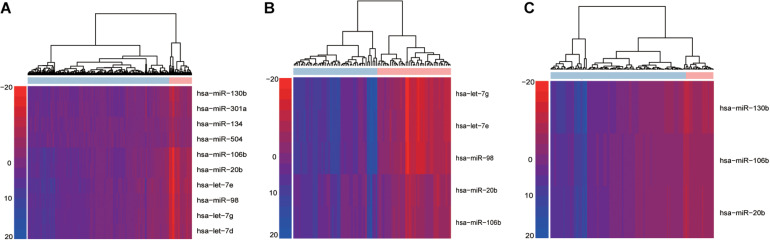
Hierarchical cluster analysis of high-frequency miRNA-mediated subpathways in *within-dataset* analyses. Based on the results of 150 classifiers, the risk biomarkers identified in the **(A)** “PRAD-TCGA,” **(B)** GSE14794, and **(C)** GSE21036 analyses were subjected to hierarchical cluster analysis. Rows and columns represent miRNA-mediated subpathways (miRNAs) and samples, respectively.

Using the “PRAD-TCGA” dataset (before going on the do the same with the GSE21036 and GSE14794 GEO datasets), we employed Equation 1 to calculate the topological weight of each gene node in the GDPN. Equations 2, 3 were used to infer the miRNA-mediated subpathway (miRNA) activity profile. The rows and columns of the activity profile matrix refer to the miRNA-mediated subpathways (miRNAs) and samples, respectively. This matrix had 220 rows (miRNAs) and 546 columns (samples). We then computed the Student’s *t*-test *p*-values for the miRNA-mediated subpathways in the activity profile and sorted them by ascending *p*-value. The top 50 miRNA-mediated subpathways were then used as candidate biomarkers and were subjected to SVM procedures using the “e1071” package in R. The number 50 was chosen based on the plateauing of the AUCs. To obtain validated risk biomarkers for prostate cancer and evaluate the performance of our method, fivefold cross-validation was performed 10 times. The AUC fluctuated between 0.848 and 0.998. We counted the frequency of each miRNA-mediated subpathway (miRNA) among the 150 SVM constructed classifiers and sorted by descending frequency. We only kept the miRNA-mediated subpathways with a frequency > 50 for further analysis, and we designated them as the high-frequency risk biomarkers. Thus, for the “PRAD-TCGA” dataset, 10 miRNA-mediated subpathways were identified as risk biomarkers. We used these 10 risk biomarkers to perform hierarchical clustering based on the miRNA-mediated subpathway activity profile (Figure 2A). Next, we obtained risk biomarkers based on the two GEO datasets and performed hierarchical clustering using their miRNA-mediated subpathway activity profiles (Figures 2B,C). Figures 2A–C show that the risk biomarkers of the various datasets could clearly separate normal and cancer samples.

The results indicated that our method could identify risk biomarkers that could be used to divide samples into normal and cancer groups (Figures 2A–C). Additionally, the risk biomarkers (miRNAs) were found to be related to prostate cancer, as indicated by data from the Human MicroRNA Disease Database (HMDD) (Supplementary Table 1-miRNAs).

### Integrating Topological Information Into the Activity Value

**FIGURE 3:**
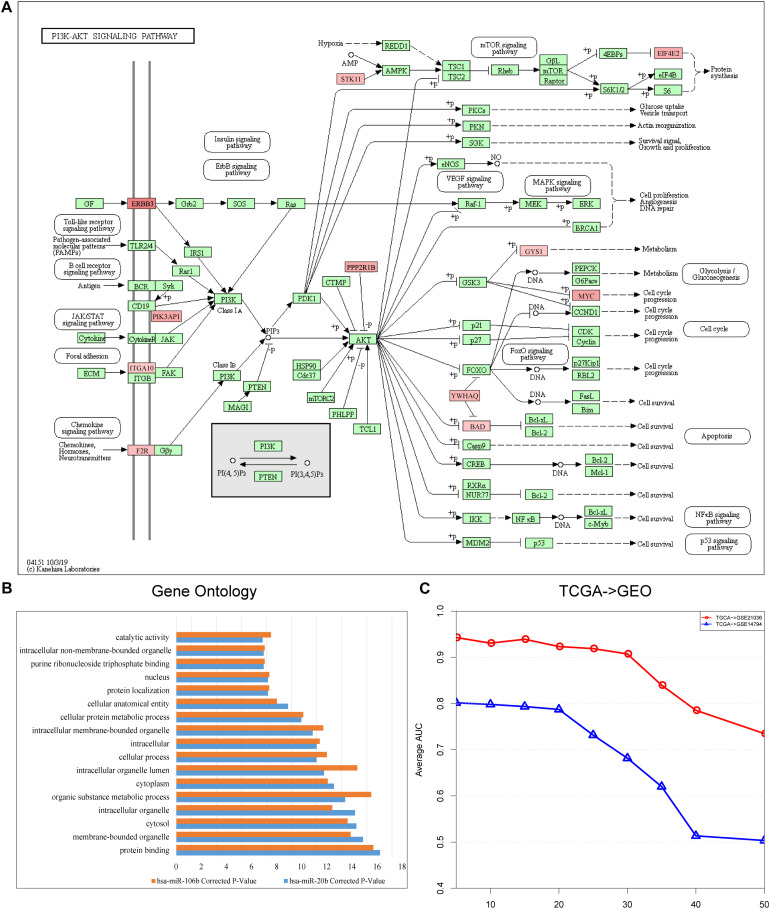
**(A)** Landscape of PI3K-Akt signaling pathway (hsa04151). Red represents the target genes of hsa-miR-106b, and the intensity of red represents the level of differential expression. **(B)** Shared Gene Ontology (GO) terms of the target genes of hsa-miR-106b and hsa-miR-20b obtained by using KOBAS 3.0. **(C)** Line graph indicating the importance of the topological information. The x-axis refers to the percentage of deleted miRNA–target gene pairs; the y-axis refers to the corresponding AUCs.

We identified risk biomarkers (miRNAs) based on the differences in miRNA-mediated subpathway activities between normal and cancer samples, which could help us to understand the biological mechanisms underlying cancer. We only considered target genes of miRNAs with *p* < 0.05 in the GDPN. The target genes of miRNAs with significantly differential expression were designated SDE target genes. To better understand the functions of the miRNA-mediated subpathways, these SDE target genes were annotated using KEGG pathways, and the pathways were sorted by ascending *p*-value. The pathways with a false discovery rate (FDR) < 0.05 and *p* < 0.01 (Benjamini and Hochberg method) were further analyzed. The SDE target genes of the 10 risk biomarkers (miRNA-mediated subpathways) in the “PRAD-TCGA” dataset were annotated with 299 KEGG pathways. Among these pathways, we selected the 41 pathways with 10 occurrences. Of these 41 pathways, 31 (82.93%) were related to prostate cancer, according to studies in PubMed (Supplementary Table 1-Pathways). The pathways associated with the SDE target genes of the 10 risk biomarkers included “Phosphatidylinositol-3-kinase (PI3K)-Akt signaling pathway” (hsa04151, Figure 3A), “mammalian target of rapamycin (mTOR) signaling pathway” (hsa04150), “mitogen-activated protein kinase (MAPK) signaling pathway” (hsa04010), and “cAMP signaling pathway” (hsa04024). The PI3K-Akt signaling pathway is a major research topic in prostate cancer treatment development, with more and more researchers paying close attention to it (Morgan et al., 2009; Toren and Zoubeidi, 2014). In prostate cancer, the activation of this pathway appears to be a characteristic of many aggressive cases, and this activation is observed more frequently as prostate cancer progresses to become a drug-resistant, metastatic disease (Toren and Zoubeidi, 2014). Many studies have shown that the PI3K-Akt signaling pathway plays a crucial role in prostate cancer metastasis and progression, along with the mTOR signaling pathway (Morgan et al., 2009; Bitting and Armstrong, 2013; Kim et al., 2017; Popolo et al., 2017). Moreover, the PI3K-Akt-mTOR and MAPK pathways can cooperate to facilitate prostate cancer growth and drug resistance (Shorning et al., 2020). Prostate cancer cell growth and invasion also involve the prostaglandin E2 receptor EP4 *via* the cAMP-PKA/PI3K-Akt signaling pathway (Xu et al., 2018). Thus, many studies have shown that oncogenic activation of the PI3K-Akt-mTOR pathway is a frequent event in prostate cancer that facilitates tumor formation, disease progression, and drug resistance (Shorning et al., 2020).

Moreover, we obtained new topological weights for the SDE target genes in the GDPN and sorted by descending weight. Among the top 100 SDE target genes (with topological importance), 22 genes were involved in the pathways with 10 occurrences regarding the SDE target genes of the 10 risk biomarkers (miRNA-mediated subpathways) in the “PRAD-TCGA” dataset. Several of the 22 genes are known to be important genes involved in prostate cancer. For example, coiled coil domain containing 6 (CCDC6) and DEAD-box RNA helicase p68 (DDX5) were annotated to “Pathways in cancer” (hsa05200) and “Transcriptional misregulation in cancer” (hsa05202), which are associated with cancer initiation and progression. Furthermore, eukaryotic translation initiation factor 4E (EIF4E) was annotated to the “PI3K-Akt signaling pathway” (hsa04151) and “mTOR signaling pathway” (hsa04150). CCDC6 protein turnover is regulated by the de-ubiquitinase USP7, which also controls androgen receptor (AR) stability (Criscuolo et al., 2019). Therefore, CCDC6 might be a predictive biomarker for the effectiveness of USP7 inhibitor and PARP inhibitor combination treatment in advanced prostate cancer (Criscuolo et al., 2019). DDX5 is an important AR transcriptional co-activator in prostate cancer and is overexpressed in late-stage disease (Clark et al., 2013). It is recruited to the AR transcriptional complex and required for the transcriptional regulation of AR-targeted genes (You et al., 2019). EIF4E plays a key role in protein synthesis and tumorigenesis (Xie et al., 2020). Regulation of EIF4E is partly achieved *via* phosphorylation (Furic et al., 2010). Ectopic expression of EIF4E prevented phenethyl isothiocyanate (PEITC)-induced translation inhibition and conferred significant protection against PEITC-induced apoptosis (Hu et al., 2007).

After discovering that hsa-miR-106b and hsa-miR-20b were the two biomarkers shared by the three datasets (“PRAD-TCGA,” GSE21036, and GSE14794 datasets), we used KOBAS 3.0 to annotate their differentially expressed target genes with Gene Ontology (GO) terms. We then extracted the shared Gene Ontology (GO) terms (Figure 3B). The results showed that the differentially expressed target genes were associated with protein binding and cellular metabolic processes.

Finally, to assess the importance of the topological structure, 10, 20, 30, 40, and 50% of the miRNA–target gene pairs were randomly deleted. Figure 3C shows that the average AUC decreased as the percentage of deleted pairs increased. However, the average AUCs of our method remained stable when we deleted only 20% or only 30% in the “TCGA–GSE14794” and “TCGA–GSE21036” analysis, respectively. Lower stability was observed in the “TCGA–GSE14794” analysis, which might be caused by the fewer samples in the cell line dataset (GSE14794). The results indicate that stable performance might be best achieved by our method of integrating multi-omics data and topological weights, allowing risk biomarkers that can robustly classify samples to be identified.

### Our Method Applied to Prostate Cancer Datasets

To compare our method with other methods, we searched PubMed for studies involving similar methods, but there were no similar methods. However, we identified five classical methods to compare with our method, and these were pathway-based methods. They were the Mean method, Median method (Guo et al., 2005), component analysis (PCA) method (Bild et al., 2006), pathway activity inference using condition-responsive genes (PAC) method (Lee et al., 2008), and a previous DRW method (Liu et al., 2013). Furthermore, another two traditional methods, which classify samples based on single molecules (genes or miRNAs) were used in the comparison. The evaluation was performed similarly to the evaluation described by Lee et al. (Hu et al., 2007), who evaluated the classification performance of miRNA-mediated subpathways by fivefold cross-validation in a *within-dataset* analysis. To ensure an unbiased comparison, the SVM models were built based on the same datasets and evaluated based on the top 50 candidate biomarkers. As mentioned, in addition to pathway-based classification methods, two traditional classifiers were established based on genes and miRNAs, respectively; these classifiers were built with genes/miRNAs that belonged to the GDPN.

**FIGURE 4:**
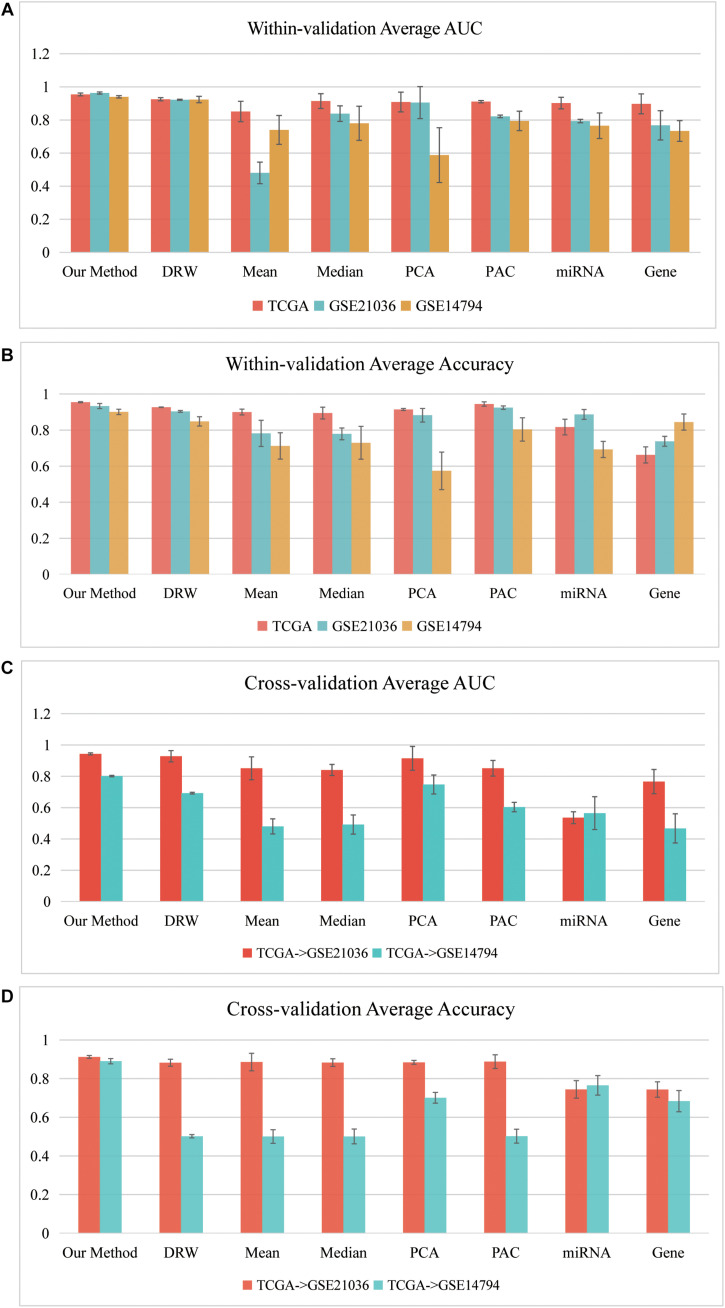
Classification performances of our SVM model in *within-dataset* analyses. **(A)** Average AUC and **(B)** average Accuracy of the eight methods, including our method, which was calculated based on 150 SVM classifiers in each *within-dataset* analysis. **(C)** Average AUC and **(D)** average Accuracy of the eight methods, including our method, which was calculated based on 50 SVM classifiers in the “TCGA–GSE21036” and “TCGA–GSE14794” *cross-dataset* analyses.

Figure 4 depicts a summary of the average AUC and Accuracy in the *within-dataset* and *cross-datasets* analyses. The average AUC (0.9525) and Accuracy (0.9296) of our method in three *within-dataset* analyses (“PRAD-TCGA,” GSE14794, and GSE21036 analyses) were calculated. We then examined the minimum standard deviation of the AUCs and Accuracies, which were 0.007 and 0.011 (Figures 4A,B), respectively. The average AUC and Accuracy outperformed the corresponding values for the pathway-based methods in the *within-dataset* analyses (Supplementary Table 1-Wilcoxon signed-rank test).

The results indicated that the miRNA-mediated subpathways could classify the sample phenotypes (normal vs. cancer). The results also revealed that our method was an effective strategy, integrating topological information into miRNA-mediated subpathway activities for sample classification. Thus, our classification biomarkers were more discriminative and stabler.

Furthermore, we performed *cross-datasets* analyses (“TCGA–GSE21036” and “TCGA–GSE14794”) using the three prostate cancer datasets. The “PRAD-TCGA” dataset was used as the *training* set, and GSE21036 and GSE14794 were used as the *test* sets. The average AUC (Accuracy) in the “TCGA–GSE21036” and “TCGA–GSE14794” analyses were 0.9434 (0.9123) and 0.8015 (0.8903), respectively. For these *cross-datasets* analyses, the average AUC (Accuracy) of our SVM model was larger than corresponding values for the pathway-based, gene-based, and miRNA-based methods (Figures 4C,D). The average AUC (Accuracy) in the “TCGA–GSE14794” analyses compared to the “TCGA–GSE21036” analyses was slightly decreased, due to the imbalance between the *training* and *test* sets. Although the three prostate cancer datasets were obtained using different sequencing platforms and patient samples, the average AUC and Accuracy associated with our method were > 0.80. Therefore, our method detected accurate classification biomarkers (miRNA-mediated subpathways), which may be useful for diagnosis, and they had strong generalization ability and classification power.

### Evaluating Our SVM Model in Another 10 Cancer Datasets

**FIGURE 5:**
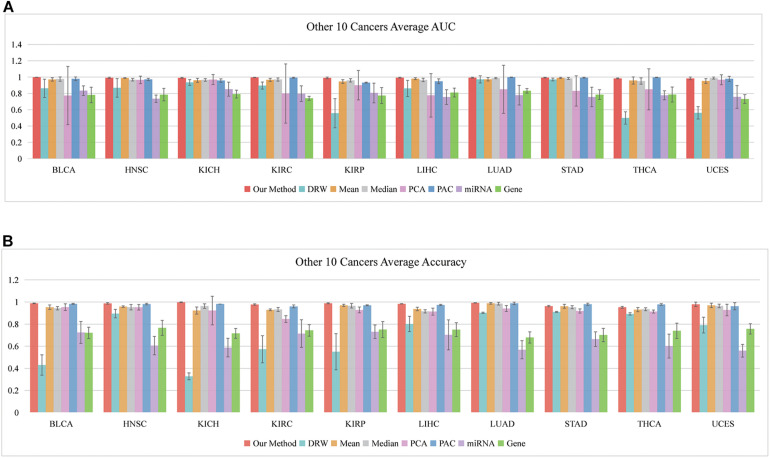
Classification performances of our SVM method based on 10 other cancers. **(A)** Average AUCs and **(B)** average Accuracies of the eight methods, including our method.

So far, we have shown that the set of classification biomarkers (miRNA-mediated subpathways) work for prostate cancers. The consistency of these biomarkers was demonstrated by evaluating the SVM model both in *within-dataset* and *cross-datasets* analyses. Nevertheless, we had to consider whether the activity profile could be used to classify samples of other cancers. A total of 31 cancer datasets were downloaded from UCSC Xena (see text footnote 1). To avoid overfitting, we subjected 10 datasets to further analysis, each of which had >200 samples and sample-matched miRNA and gene expression profiles. We performed 10 fivefold cross-validation experiments on the 10 datasets (150 AUCs and Accuracies were calculated) and obtained the average AUCs and Accuracies (Figures 5A,B). To ensure an unbiased evaluation, the activity profiles in the 10 datasets were calculated to build the classifiers, and the frequency of each miRNA-mediated subpathway in each cancer was counted. The miRNA-mediated subpathways with frequency >50 were used as risk biomarkers for each cancer. A total of 56 risk biomarkers were used for further analysis, and most of them were related to specific cancers. Only eight miRNA-mediated subpathways occurred in >5 cancers, while 19 occurred in a single cancer only. The result implied that our method could detect cancer-specific risk biomarkers (miRNAs).

For example, hsa-let-7c, hsa-let-7i, hsa-let-7b, and hsa-let-7g occurred in seven, six, five, and five, respectively. Overwhelming evidence has demonstrated that the miRNA let-7 family (−a, −b, −c, −d, −e, −f, −g, and −i) plays regulatory roles at the transcriptional and post-transcriptional levels among various species, and their aberrant expression might be closely linked to the pathogenesis of cancers (Gibadulinova et al., 2020; Liu et al., 2020). According to our method, hsa-miR-191 appeared 130 times in the frequency list of liver hepatocellular carcinoma (LIHC), and it has been reported to play an important role in hepatocellular carcinoma (HCC) (Tian et al., 2019). Its overexpression reversed the anti-tumor effect of ANRIL on HepG2 cell proliferation, apoptosis, migration, and invasion (Huang et al., 2018). The hsa-miR-141 appeared in kidney renal clear cell carcinoma (KIRC) with high frequency, and it may play a crucial role in the diagnosis of kidney carcinoma. It also robustly discriminated between malignant and non-malignant tissues, and inhibiting it in normal renal proximal tubule epithelial cells (RPTEC) induced pro-cancerous characteristics (Dasgupta et al., 2020). It also acted as a potential biomarker for discriminating renal clear cell carcinoma (ccRCC) from normal tissues, and it acted as a crucial suppressor of ccRCC cell proliferation and metastasis by modulating the EphA2/p-FAK/p-AKT/MMPs signaling cascade (Chen et al., 2014).

The external evaluation in the 10 cancer datasets implied that practical risk biomarkers (miRNA-mediated subpathways) could be detected using our method. Moreover, the key target genes of the identified miRNAs were located in multi-pathway core regions. Identification of these regions provides opportunities to explore the interactions among genes, miRNAs, and pathways during cancer development.

### Case Study

Applying our method to the “PRAD-TCGA” dataset, we obtained 10 miRNA-mediated subpathways (miRNAs) and their 721 differently expressed target genes. The miRNA-mediated subpathway activity profile was inferred based on the SDE target gene expression and topological weights. Thus, the risk biomarkers (miRNA-mediated subpathways) had a stronger classification capacity.

We identified the shared miRNA-mediated subpathway biomarkers by comparing the results between the “PRAD-TCGA,” GSE21036, and GSE14794 datasets. The hsa-miR-106b and hsa-miR-20b were the biomarkers that were shared among the three datasets. For the former miRNA, there were 273 miRNA–SDE target gene pairs, and for the latter, there were 277. The SDE target genes of these two miRNAs were annotated to several pathways, including “Metabolic pathways” (hsa01100) and “Protein processing in endoplasmic reticulum” (hsa04141). These pathways were ranked first and second, respectively, in the pathway list based on *p*-values (FDR < 0.05, Benjamini and Hochberg method). Previous gene expression research has shown that metastasis in prostate cancer is related to metabolic pathway dysregulation (Rhodes et al., 2002) and dysregulated transcriptional programs (LaTulippe et al., 2002). Research has also shown that, in androgen-independent prostate cancer cells, several small-molecule modulators of Sigma1 altered the endoplasmic reticulum (ER)-associated protein homeostasis pathways, including the unfolded protein response and autophagy (Maher et al., 2018). Furthermore, the SDE target genes of the two abovementioned miRNAs were also annotated to the “TGF-beta signaling pathway” (hsa04350), which was one of the top 10 pathways in the pathway list. Research has shown that induction of miR-106b plays a crucial role in the suppression of the proliferation of prostate cancer cells in a process that involves the TGF-beta signaling pathway (Zhang et al., 2012). Additionally, in human prostate cancer, miR-20b targets and downregulates TGFBR2, which in turn affects Smad2 activation and E2F1 expression, dysregulating the miR-20b-5p expression and contributing to TGF-β-induced epithelial-to-mesenchymal transition (Qi et al., 2019).

Notably, several miRNAs (such as hsa-miR-98, which was identified as a biomarker in the “PRAD-TCGA” analysis, and hsa-miR-301a, which was identified as a biomarker in both the “PRAD-TCGA” and GSE14794 analyses) play important roles in prostate cancer by regulating their target genes and thereby regulating pathways related to cancer. For example, hsa-miR-98 (which was annotated to 186 pathways) targeted nine differentially expressed genes in the “TGF-β signaling pathway” (hsa04350; E2F5, CDKN2B, MYC, BMP6, ACVR2B, SMAD7, ZFYVE16, RPS6KB2, and CHRD), and TGF-β affects multiple cellular responses *via* the canonical SMAD pathway and noncanonical pathways like the MAPK and PI3K-AKT pathways (Hamidi et al., 2017). Regarding E2F5, the E2F5/p38 axis plays a major role in uncontrolled prostate cancer cell proliferation *via* pSMAD3L activation, which provides strong support for using E2F5 as a biomarker for early detection of prostate cancer (Majumder et al., 2016). Additionally, regarding CDKN2B, upregulation of inhibitor of differentiation (Id1 and Id3) proteins attenuates all three cyclin-dependent kinase inhibitors (CDKN2B, -1A, and -1B), resulting in a more aggressive prostate cancer phenotype (Sharma et al., 2012). Moreover, regarding MYC, MYC-regulated fatty acid synthesis has been reported to be a valid target for treatment and/or prevention of prostate cancer. Not only are these three genes (E2F5, CDKN2B, and MYC) known to be associated with prostate cancer, but the six other target genes of hsa-miR-98 (BMP6, ACVR2B, SMAD7, ZFYVE16, RPS6KB2, and CHRD) have also been reported to be relevant to prostate cancer, according to previous studies (Supplementary Table 1-Genes). Moreover, hsa-miR-301a was annotated to the “p53 signaling pathway” (hsa04115), which can play a key role in the effectiveness of certain prostate cancer treatments. The ATM-CHEK2-p53 axis acts as a backbone for the DNA damage response (DDR) and is hypothesized to act as a barrier to cancer initiation (Stolarova et al., 2020). Significant associations with familial prostate cancer risk have been reported for both CHEK2 and ATM (Wokolorczyk et al., 2020).

The case study indicated that our SVM model could identify the key miRNAs, which may be useful as classification biomarkers for prostate cancer.

## Discussion

Prostate cancer is a complicated cancer that has a high level of heterogeneity, many symptoms, and multiple subtypes. In fact, prostate cancer has a lack of clear classification biomarkers because of its high heterogeneity and molecular instability. Clinically, age, prostate-specific antigen level, and Gleason score are generally used to diagnose this cancer among males. The miRNAs in blood and urine represent a convenient source of biomarkers for prostate cancer diagnosis and assessment of treatment efficacy due to their high stability and the low invasiveness of the sample collection process (Konoshenko et al., 2020).

Using machine learning to identify risk classification biomarkers of prostate cancer is a challenging task. With the increasing amount of high-throughput sequencing data, more and more miRNAs that are closely related to prostate cancer, such as hsa-miR-134 (Pelka et al., 2020), hsa-miR-504 (Katoh et al., 2013), and the hsa-let-7 family (Liu et al., 2012; Rong et al., 2020), have been discovered. Many researchers are now putting effort into identifying robust miRNA biomarkers of prostate cancer. However, there are no previously published sets of specific miRNA biomarkers for classifying samples into normal and prostate cancer groups, and the results of studies on miRNA biomarkers in other cancers often report conflicting results.

Due to advances in high-throughput multi-omics technologies, integrating multi-omics data into a special score is a promising approach to identifying biomarkers that can classify normal and cancerous samples. This integration strategy eliminates the dependence of machine learning on single types of data, such as gene or miRNA expression data. Thus, the approach provides an opportunity to detect robust classification biomarkers.

As is well known, the causes of cancers are complicated. Some researchers are convinced that biological pathways are disrupted by the target genes of miRNAs. Moreover, the target genes of single miRNAs can be found in several pathways. Pathways, miRNAs, and their target genes are involved in the occurrence, development, and metastasis of cancer, with the miRNAs playing a bridging role between pathways and genes. Also, many researchers believe that disease phenotypes are highly related to key local subpathways, rather than entire pathways (Li et al., 2013). We hold the opinion that our method is a promising way to detect classification biomarkers and to understand the biological mechanisms of cancer. The topological structure of biological networks should be considered when identifying risk biomarkers.

The purpose of our study was to identify robust classification biomarkers, and our method precisely classified samples. The method involved five steps: merge pathways and construct network; perform DRW; infer miRNA-mediated subpathway activity; select features and evaluate classification method; and obtain risk biomarkers. Each classification biomarker was composed of multiple types of integrated data, which included the GDPN topological information and the expression levels of miRNAs and their target genes. We reassigned new topological weights to the gene nodes using a DRW-based method. More topological weight was assigned to the gene nodes that exhibited topological importance. We amplified the signal of dysregulated hub genes and reassigned them larger topological weights because the expression of hub genes tends to vary only weakly between cases and controls (Lu et al., 2007). The miRNA-mediated subpathway activities were robust, and they may lead to better classification.

## Data Availability Statement

The original contributions presented in the study are included in the article/Supplementary Material, further inquiries can be directed to the corresponding author/s.

## Author Contributions

ZN and XY proposed and designed the method pipeline. ZN downloaded and filtered all datasets, performed the training process, and drafted the manuscript. SY, YZ, XS, HW, and XY revised the manuscript. All authors carefully examined and analyzed all datasets. The manuscript was reviewed by all authors.

## Conflict of Interest

The authors declare that the research was conducted in the absence of any commercial or financial relationships that could be construed as a potential conflict of interest.

## Footnotes

1 https://xena.ucsc.edu/

2 http://www.ncbi.nlm.nih.gov/geo/

3 https://github.com/chunquanlipathway

## Supplementary Material

Supplementary Table 1 Details of results based on our SVM method. Genes: 22 key genes among the top 100 differentially expressed genes with high topological importance, and associated studies. miRNAs: High-frequency biomarkers (miRNA-mediated subpathways), and associated studies. Pathways: High-frequency pathways (with 10 occurrences) that the miRNA-mediated subpathways were annotated with, and associated studies. Wilcoxon signed-rank test: Wilcoxon signed-rank test results regarding the *within-dataset* and *cross-datasets* AUCs showing that our method outperformed the previous methods.
